# Genome-Wide Identification of MAPK, MAPKK, and MAPKKK Gene Families in *Fagopyrum tataricum* and Analysis of Their Expression Patterns Under Abiotic Stress

**DOI:** 10.3389/fgene.2022.894048

**Published:** 2022-06-17

**Authors:** Zhen Wang, Song Yan, Weichao Ren, Yan Liu, Wei Sun, Meiqi Liu, Jiaxin Lu, Yaolei Mi, Wei Ma

**Affiliations:** ^1^ Pharmacy of College, Heilongjiang University of Chinese Medicine, Harbin, China; ^2^ Institute of Chinese Materia Medica, China Academy of Chinese Medical Sciences, Beijing, China; ^3^ Key Laboratory of Basic and Application Research of Beiyao (Heilongjiang University of Chinese Medicine), Ministry of Education, Harbin, China

**Keywords:** *Fagopyrum tataricum*, MAPK cascade, genome-wide, expression patterns, abiotic stress, co-expression

## Abstract

The mitogen-activated protein kinase (MAPK) cascade is a highly conserved signal transduction pathway, ubiquitous in eukaryotes, such as animals and plants. The MAPK cascade has a dominant role in regulating plant adaptation to the environment, such as through stress responses, osmotic adjustment, and processes that modulate pathogenicity. In the present study, the MAPK cascade gene family was identified in *Fagopyrum tataricum* (Tartary buckwheat), based on complete genome sequence data. Using phylogenetic tree, conservative motif, and chromosome location analyses, a total of 65 FtMAPK cascade genes, distributed on five chromosomes, were classified into three families: MAPK (n = 8), MAPKK (n = 1), and MAPKKK (n = 56). Transcriptome data from Tartary buckwheat seedlings grown under different light conditions demonstrated that, under blue and red light, the expression levels of 18 and 36 FtMAPK cascade genes were up-regulated and down-regulated, respectively. Through qRT-PCR experiments, it was observed that FtMAPK5, FtMAPKK1, FtMAPKKK8, FtMAPKKK10, and FtMAPKKK24 gene expression levels in the Tartary buckwheat seedlings increased under three types of abiotic stress: drought, salt, and high temperature. A co-expression network of FtMAPK cascade genes was constructed, based on gene expression levels under different light conditions, and co-expressed genes annotated by Gene Ontology and Kyoto Encyclopedia of Genes and Genomes enrichment analyses, which identified numerous transcription factors related to plant abiotic stress. The authors conclude that FtMAPK cascade genes have important roles in the growth and development of Tartary buckwheat, as well as its responses to abiotic stress.

## Introduction

Phosphorylation is an important post-translational modification (PAM), which is the main mechanism underlying signal transduction (Shiu et al., 2004). Protein kinases are enzymes that catalyze the phosphorylation of proteins, and protein phosphorylation can control enzyme activity and regulate interactions among molecules (Johnson and Lapadat, 2002), to mediate and amplify signal transmission. Phosphorylation of proteins is involved in the intracellular transmission of various stimulatory signals, such as plant hormones, including abscisic acid, ethylene, cytokinin, and auxin, or light, salt stress, drought stress, pathogen infection, and other external environmental stimuli (Wang et al., 2020). Three types of protein kinases have been isolated from plants: tyrosine, histidine, and serine/threonine-protein kinases. These are classified according to the different amino acid residues phosphorylated in protein substrates. The genes encoding the serine/threonine-protein kinase family of mitogen-activated protein kinase (MAPK) molecules comprise the most extensively studied gene family (He et al., 2020a).

Plant growth and development are severely restricted by abiotic stress. Approximately 50% of annual global yield losses of major crops are related to abiotic stress (Valliyodan and Nguyen, 2006). To ensure normal growth and development, plants have evolved various stress response mechanisms during their long evolutionary history, among which the recognition and transmission of stress signals and their amplification and transduction are key factors that activate response signals to resist the damage caused by stress (Xiong et al., 2002). Therefore, the study of the signal transduction mechanisms, underlying plant stress responses and screening for genes that can enhance the resistance of plants to stress, is of great significance. In higher plants, the MAPK cascade pathway is highly evolutionarily conserved and both affects plant growth and influences hormone regulation, and biotic and abiotic stress responses (Yanagawa et al., 2016).

The coarse grain crop, Tartary buckwheat (*Fagopyrum tataricum*), is characterized by cold and frost resistance, tolerance to arid conditions, self-pollination, a short growth cycle, and a high seed-setting rate (Lauranne et al., 2021). Tartary buckwheat originated from the arid or semi-arid Yunguichuan region of China and plateau regions of Tibet and has a long history of cultivation (Yuan et al., 2020). During its growth and development, Tartary buckwheat encounters numerous extreme environmental stimuli.

The MAPK cascade plays an important role in regulating processes involved in plant adaptation to the environment, and this has been confirmed in numerous plant species. A high-quality Tartary buckwheat genome has been reported (Zhang et al., 2017), however, there have been no reports of the MAPK, MAPKK, and MAPKKK gene families of Tartary buckwheat. In the current study, we generated new data regarding the Tartary buckwheat MAPK gene family, to support classification and functional analyses.

## Materials and Methods

### Identification and Analysis of the Physicochemical Properties of MAPK Cascade Genes in the Tartary Buckwheat Genome

The *F. tataricum* (GCA_002319775.1), *Arabidopsis thaliana* (GCA_000005425.2), *Solanum lycopersicum* (GCF_000188115.4), *Cucumis sativus* (GCF_000004075.3), *Malus domestica* (GCF_002114115.1), *Vitis vinifera* (GCF_000003745.3), and *Fragaria vesca* (GCF_000184155.1) whole-genome sequences and annotation files were downloaded from the NCBI website (https://www.ncbi.nlm.nih.gov/). TBtools software (Chen et al., 2020) was used to generate a local Arabidopsis MAPK cascade genes database, and Tartary buckwheat MAPK cascade gene sequences were identified using the BLAST application against the Arabidopsis dataset. Then, BLAST searches were conducted through the NCBI website, to confirm the sequences and reduce redundancy. Protein sequences corresponding to identified genes were extracted, to identify all Tartary buckwheat MAPK protein sequences. The ExPASy online tool (http://web.expasy.org/protparam/) was used to predict the physical and chemical properties of Tartary buckwheat MAPK cascade proteins.

### Gene Structure, Conserved Motifs, and Phylogenetic Tree Analysis of MAPK Cascade Proteins

TBtools software was used to analyze MAPK cascade protein sequences and genomic annotation files, to generate gene structure maps. The MEME website (http://memesuite.org/tools/meme) was employed to mine for conserved motifs, using annotation of zero or one occurrence per sequence. Phylogenetic trees of Tartary buckwheat and *A. thaliana* MAPK cascade proteins were constructed by adopting the Neighbor-Joining method in Mega-X software (Sudhir et al., 2018). The CLUSTALW algorithm, with default parameters, was used to generate multiple amino acid sequence alignments (the bootstrap value was set to 1000 replicates, and default values were used for other settings). Data were visualized using TBtools software.

### Chromosome Location and Gene Duplication of MAPK Cascade Genes in Tartary Buckwheat

TBtools software was used to explore the Tartary buckwheat genome annotation file, map the amino acid sequences of FtMAPK cascade genes to the genome of Tartary buckwheat, determine chromosome location information for each FtMAPK cascade gene, and draw corresponding chromosome physical location maps. MCscan (Wang et al., 2012) was used to identify FtMAPK cascade gene duplication events. TBtools software was also used to analyze the collinear relationships between Tartary buckwheat and six other plant genomes, focusing on MAPK cascade genes.

### Analysis of *FtMAPK* Cascade Gene *cis-*Elements

TBtools software was used to extract 2000 bp of sequence upstream of each FtMAPK gene as promoter regions (Zhang et al., 2021), which were then submitted to the promoter region sequence PlantCRAE online website (http://bioinformatics.psb.ugent.be/webtools/plantcare/html/) for analysis and visualization of *cis-*elements.

### Plant Material and Abiotic Stress Treatment

Tartary buckwheat seeds used in this study were the BT18 variety from Xianning City, Guizhou Province, China. For light treatment, full-grained Tartary buckwheat seeds without damage or mildew were chosen. They were grown for 4 days in a dark environment at 25°C, then again in the dark environment, or under blue (wavelength, 470 nm) or red (wavelength, 670 nm) light, for 48 h. Treatment under dark for 6 h or UV-B for 6 h was also performed. For abiotic stress treatment, plants were first grown for 4 days at 25°C, under a 16/8 h light/dark cycle, then treated at 40°C,100 mM NaCl and 20% PEG6000 for 24 h, respectively (He et al., 2020b). The obtained Tartary buckwheat seedling samples were then frozen in liquid nitrogen and stored at –80°C. Three biological replicates were conducted for all treatments.

### Analysis and Validation of RNA-Seq Data

RNA-Seq was applied to explore the expression patterns of MAPK cascade genes in Tartary buckwheat seedlings under different light conditions. Techniques that were used for RNA extraction, isolation, sequencing, and data analysis are described in our previous report (Dong et al., 2018). TBtools software was used to construct a heat map of MAPK cascade gene expression for visual analysis, and log2 (fragments per kilobase of exon per million mapped fragments (FPKM)+1) logarithmic transformation processing was conducted to generate FPKM values.

To elucidate the expression patterns of MAPK cascade genes under different light conditions and drought stress, and to verify the results of RNA-seq data, 15 MAPK cascade genes were randomly selected and their relative gene expression levels were investigated by qRT-PCR. MAPK cascade gene sequences were used as templates for RT-PCR analysis, and specific primers were designed. The relative expression levels of each gene were calculated using the 2^-△△^ Ct method. To control the amount of template in each reaction, *Histone3* (GenBank No: JF769134.1) was used as an internal reference gene (Zhang et al., 2017). Each experiment included three technical replicates. The primers used in this study are listed in Supplementary Table 9. Raw qPCR data are provided in Supplementary Table 10.

### Protein–Protein Interaction (PPI) Network Construction

Protein interactions of Tartary buckwheat MAPK molecules were predicted using the String online tool (https://string-db.org/cgi/input.pl), with the Arabidopsis protein database selected as the reference and default parameter settings. Results were saved in the TSV format and imported into Cytoscape 3.8.0 software (Otasek et al., 2019) for visualization.

### Co-Expression Analysis and GO Enrichment Annotation of *FtMAPK* Cascade Genes

Transcription factors were identified from the whole Tartary buckwheat genome using the online database, PlantTFDB (http://planttfdb.gao-lab.org/) (Tian et al., 2019). The expression levels of all genes in Tartary buckwheat were analyzed using a Python script, and transcription factors co-expressed with MAPK cascade genes were extracted. The gene co-expression network was visualized using Cytoscape V3.8.0 software. Gene Ontology (GO) and Kyoto Encyclopedia of Genes and Genomes (KEGG) annotation were conducted using the eggNOG database (http://eggnog-mapper.embl.de/); and GO and KEGG enrichment analyses of transcription factors co-expressed with MAPK cascade genes were performed using the R package clusterProfiler (Yu et al., 2012; Wu et al., 2021).

## Results

### Identification and Characterization of *FtMAPK* Genes

**FIGURE 1:**
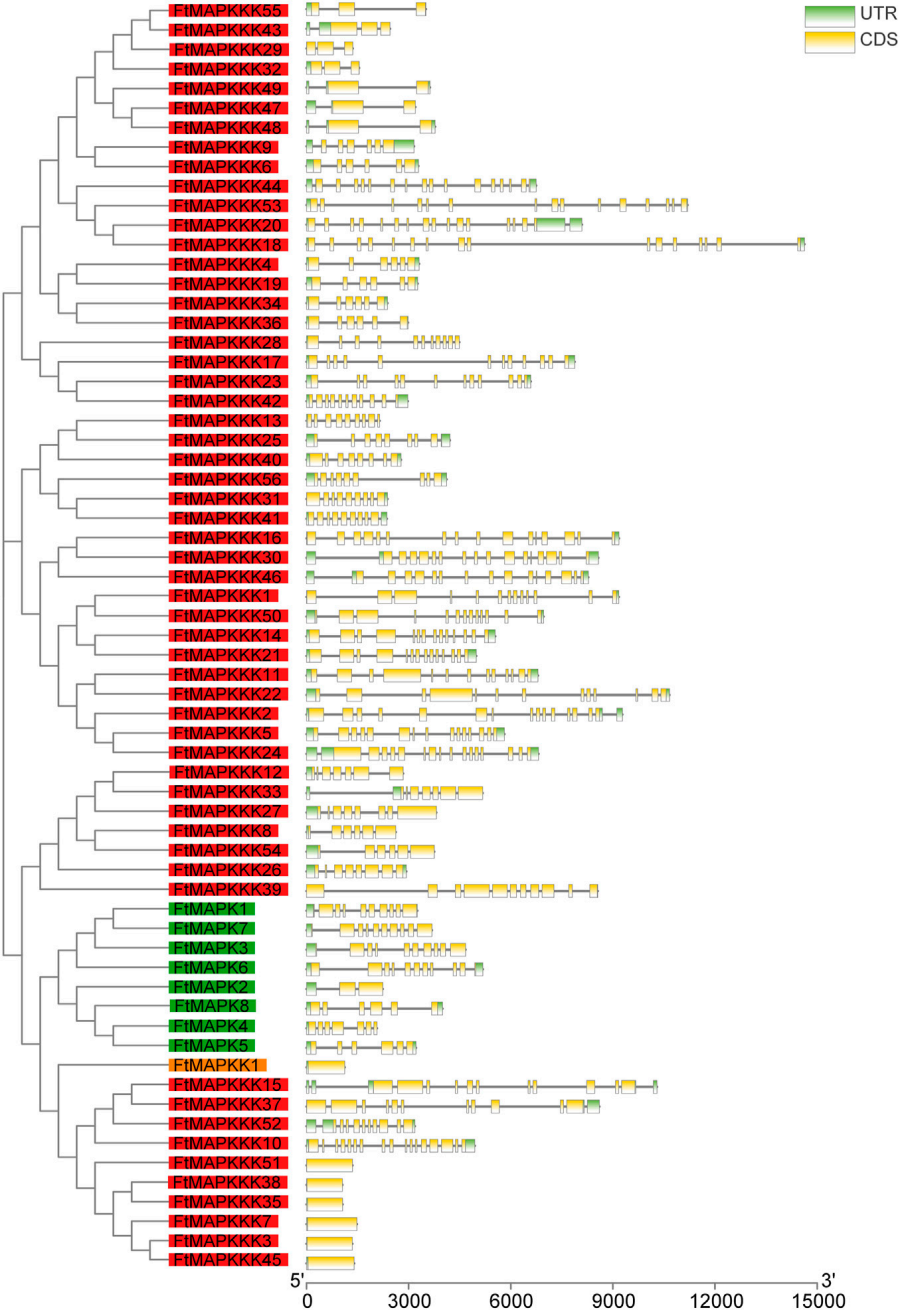
Structure of the FtMAPK cascade genes in Tartary buckwheat. The gene names on the left are arranged according to the phylogenetic tree and divided by different color background, green for the MAPK family, orange for the MAPKK family, and red for the MAPKKK family. UTR, untranslated regions; CDS, coding sequence. Introns are represented by black lines.

In common with the majority of model and horticultural plants, MAPK cascade genes in Tartary buckwheat comprise MAPK, MAPKK, and MAPKKK families. We identified a total of 65 MAPK cascade gene family members in the Tartary buckwheat genome, including 8 MAPK family members, 1 MAPKK gene, and 56 MAPKKK genes. According to their chromosomal positions, the obtained gene sequences were labeled FtMAPK1–8, FtMAPKK1, and FtMAPKKK1–56. The length of the coding sequences of the 65 MAPK cascade genes ranged from 939 to 1962 bp, encoding proteins of 312–644 amino acids, with molecular weight and theoretical isoelectric point values ranging from 35,767.46 to 124,648.9 Da and 4.73–9.51, respectively (Supplementary Table 1). The number of introns present in MAPK cascade genes ranges from 0 to 16, with seven genes lacking introns (one MAPKK family member and six MAPKKK family members) (Figure 1).

### Phylogenetic Analysis of MAPK Cascade Proteins in Tartary Buckwheat and Arabidopsis

**FIGURE 2:**
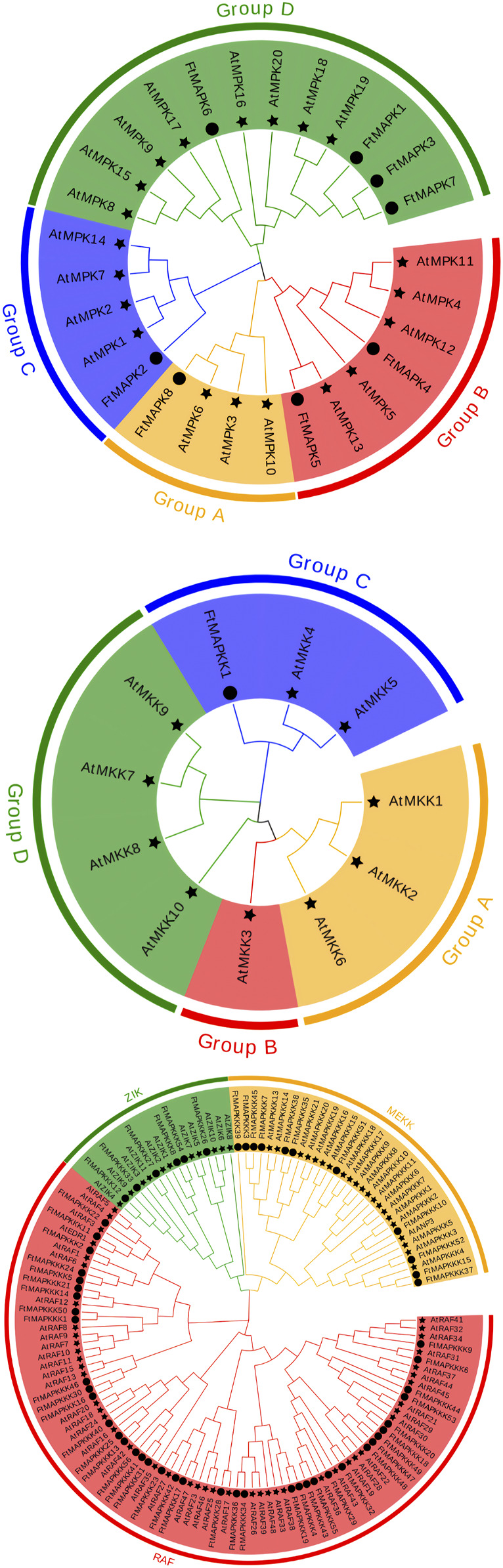
Phylogenetic tree analysis of MAPK cascade proteins from Tartary buckwheat and Arabidopsis. **(A)** refers to phylogenetic trees of MAPK family; **(B)** refers to phylogenetic tree of MAPKK family; **(C)** refers to phylogenetic tree of the MAPKKK family. The star represents *A. thaliana*, and the circle represents *F. tatpharicum*.

To better comprehend the evolutionary relationships among Tartary buckwheat MAPK cascade proteins, we downloaded and classified Arabidopsis MAPK cascade protein sequences for comparison with those in Tartary buckwheat, according to amino acid sequence similarity (Supplementary Table 2). Tartary buckwheat MAPK proteins were divided into groups A, B, C, and D. Group A included one protein (FtMAPK8), which has high homology to AtMPK6; group B had two members, of which FtMAPK5 has high homology to AtMPK6; group C also had one member; and group D had the most members (four) (Figure 2A). According to the Arabidopsis classification of MAPKK proteins into groups A, B, C, and D, the single Tartary buckwheat MAPKK protein was clustered into group C (Figure 2B). Finally, Tartary buckwheat MAPKKK proteins were divided into three categories: the ZIK, MEKK, and RAF subfamilies. Among these, the RAF subfamily had the most members (n = 39), while the MEKK subfamily had 11 members, and the ZIK subfamily had the fewest members (n = 6) (Figure 2C).

### Analysis of FtMAPK Cascade Protein Conserved Motifs

**FIGURE 3:**
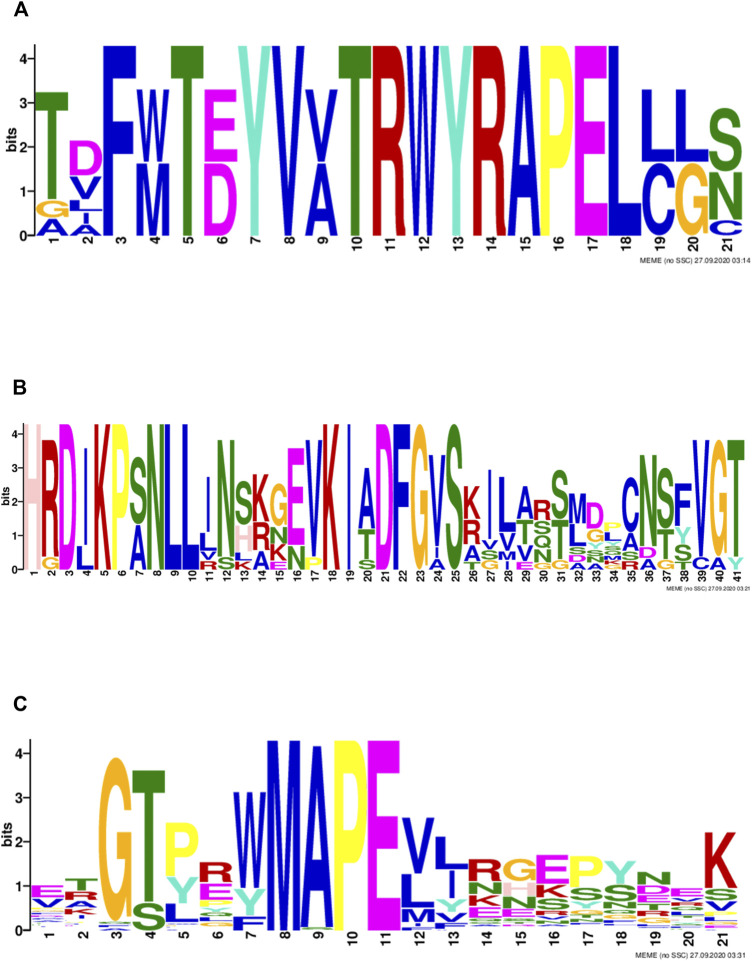
Motifs of MAPK cascade proteins in Tartary buckwheat **(A)**: the conservative motifs in MAPK protein; **(B)**: the conserved motif in MAPKK protein; **(C)**: the conserved motif in MAPKKK protein.

MAPK family genes were classified based on characteristic sequence motifs. To further explore the characteristics of MAPK cascade molecule sequences in Tartary buckwheat, we analyzed their sequences for conserved motifs (Figure 3). A conserved TD/EY motif was detected in Tartary buckwheat MAPK proteins (Figure 3A), which is the site of MAPK phosphorylation by MAPKK proteins. Further, an S/T-X5-S/T motif, representing the MAPKKK phosphorylation site in MAPKK family proteins, was detected (Figure 3B). In the MAPKKK family, an individual conserved motif was detected in each of the three subfamilies: a G (T/S)Px (W/Y/F)MAPEV motif in the MEKK subfamily, a GTXX (W/Y)MAPE motif in the RAF subfamily, and a GTPEFMAPE (L/V)Y motif in the ZIK subfamily (Figure 3C).

### Chromosomal Localization and Multi-Species Collinearity Analysis of FtMAPK Cascade Genes

**FIGURE 4:**
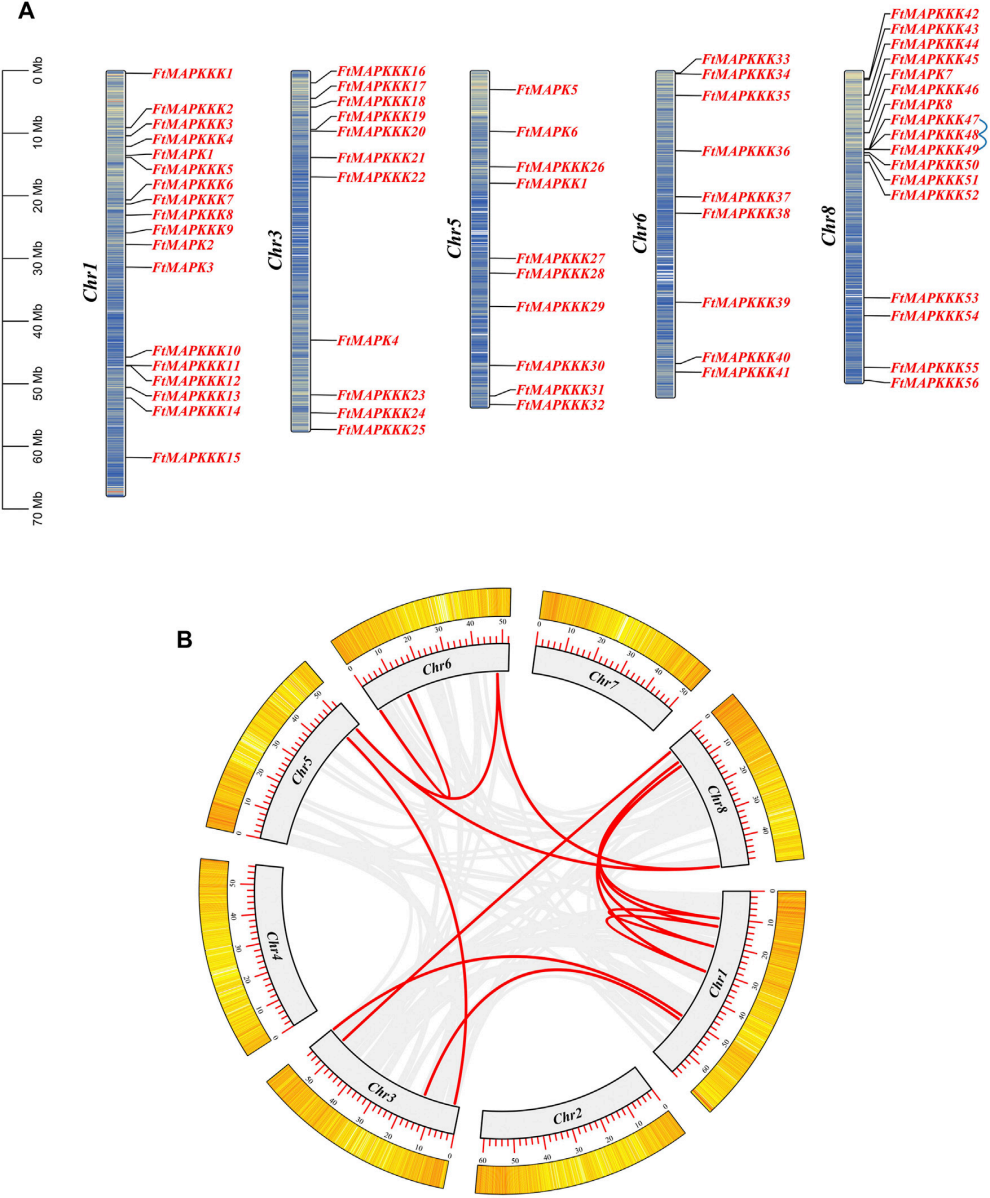
Chromosome distribution and gene replication events of FtMAPK cascade genes genes. **(A)**: Location information of FtMAPK cascade genes on Tartary buckwheat chromosome. The blue line represents pairs of tandem repeats. **(B)**: Gene segmental duplication events on the chromosome of Tartary buckwheat. The red line indicates the repeated FtMAPK gene pair, and the gray line indicates all the collinear pairs in the genome of the Tartary buckwheat.

The 65 MAPK cascade genes identified in Tartary buckwheat were found to be distributed on five chromosomes, with no clear distribution rules (Figure 4A). The largest number of genes (n = 17) was on chromosome 1, with the fewest genes distributed on chromosome 6 (n = 9, comprising only members of the MAPKKK family). Members of the MAPK, MAPKK, and MAPKKK families all map to chromosome 5.

Further analysis identified intragenomic replication events involving MAPK cascade genes. Three genes were involved in a pair of gene duplication events on chromosome 8 (Figure 4A). Further, 12 segmental replication events were detected among the 5 chromosomes (Figure 4B; Supplementary Table 3).

**FIGURE 5:**
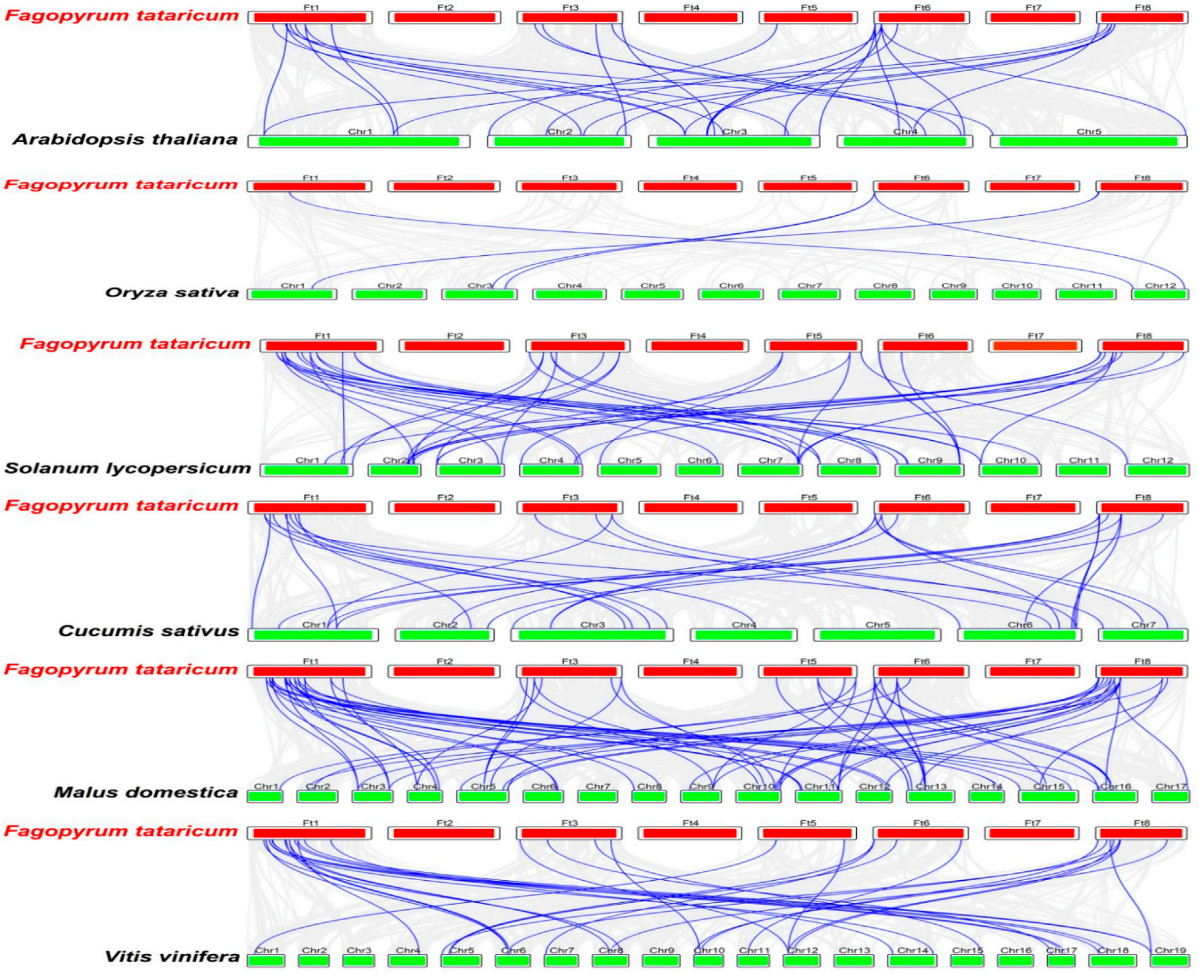
Collinearity analysis of FtMAPK cascade genes of Tartary buckwheat and other six plants. The blue line represents the collinearity between FtMAPK cascade genes and other species, and the gray line denotes the collinearity between Tartary buckwheat and all genes of other species.

To further investigate the mechanisms involved in the evolution of FtMAPK cascade genes in Tartary buckwheat and explore their similarity to related genes from other species, we selected six species (five dicotyledonous plants: *A. thaliana*, *S. lycopersicum*, *V. vinifera*, *C. sativus*, and *M. domestica*, and a monocotyledonous plant: *Oryza sativa*), with reported MAPK cascade genes, and analyzed their collinearity relationships with the Tartary buckwheat genome (Figure 5). The collinear relationship between Tartary buckwheat and A. thaliana was consistent with the results of phylogenetic analysis, and Tartary buckwheat showed a high level of collinearity with the five dicotyledonous plants, among which, the collinearity relationship with apple was strongest, while that with the monocotyledonous plant (rice) was weakest. Some genes were detected in all six plants, including *FtMAPKKK33* and *FtMAPKKK42*.

### Analysis of *Cis-*Elements in the Promoters of FtMAPK Cascade Genes

**FIGURE 6:**
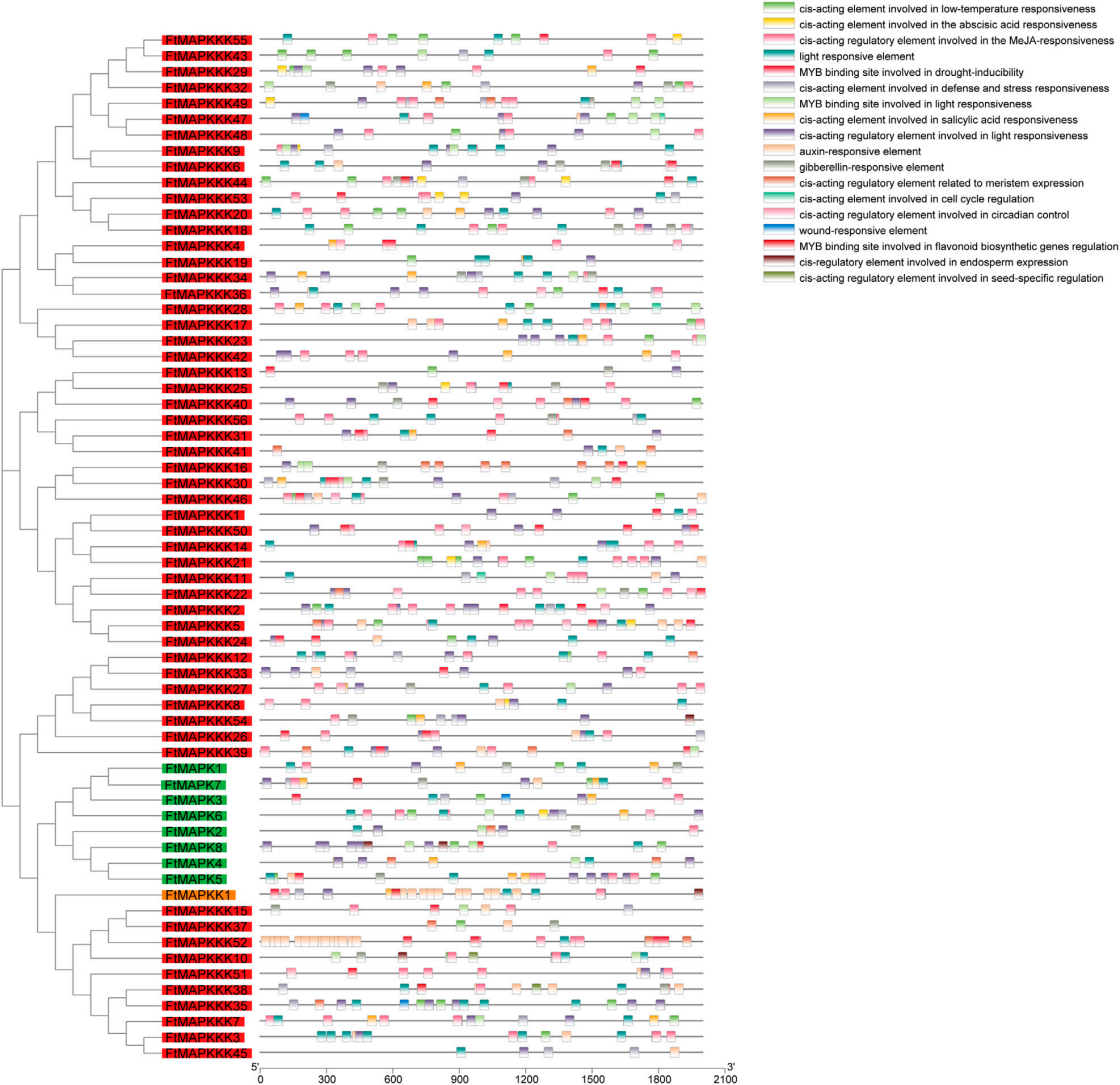
Cis-element results in promoter region of FtMAPK cascade genes. Gene names are arranged according to the phylogenetic tree and distinguished by color and divided by color, with green for the MAPK family, orange for the MAPKK family, and red for the MAPKKK family. Different colored shapes denote different cis-element. Different color rectangles represent different cis components.

*Cis-*elements are located in non-coding DNA sequences, including gene promoters, and their roles involve regulation of the transcription of related genes (Yongguan et al., 2021). In this study, we extracted the sequences 2000 bp upstream of each FtMAPK cascade gene, as their promoter regions, and used the PlantCARE web tool (http://bioinformatics.psb.ugent.be/webtools/plantcare/html/) to predict their *cis-*elements (Supplementary Table 4). We found that the cis-elements of FtMAPK cascade genes were associated with plant growth, hormones, and abiotic stress responses (Figure 6). Elements related to plant growth included circadian rhythm control, light response, and meristem expression elements, with 94% of genes containing light response elements. Hormone-related motifs included elements involved in responses to abscisic acid, salicylic acid, and methyl jasmonate; 88% of genes contained methyl jasmonate response elements. *Cis-*elements related to abiotic stress included low-temperature response and drought-induction elements, such as the MYB-binding site, indicating that MYB may regulate FtMAPK cascade genes to enhance the drought resistance of Tartary buckwheat.

### Analysis of FtMAPK Cascade Gene Expression Patterns Under Different Light Conditions

**FIGURE 7:**
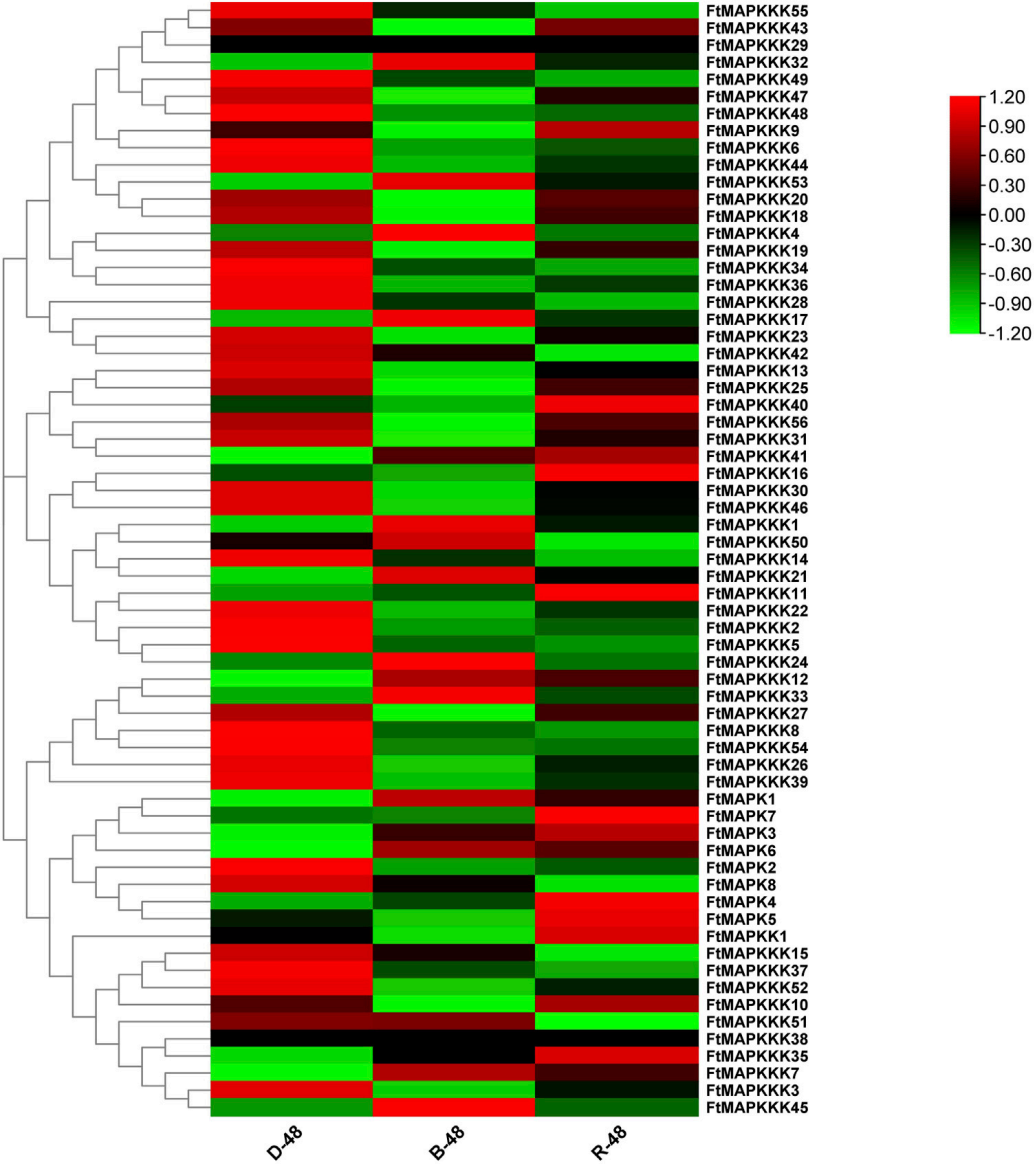
**(A)**: Analysis of FtMAPK cascade genes expression patterns in Tartary buckwheat seedlings under different light conditions. Gene expression using Log2 (FPKM+1) logarithmic transformation treatment. Red indicates high gene expression and green indicates low gene expression.

To explore the expression patterns of MAPK cascade genes in Tartary buckwheat seedlings under different light conditions, we compared their expression levels in plants grown in the dark or two kinds of light (blue and red) using RNA-seq data (Figure 7A) (Supplementary Table 5), with the aim of clarifying gene function. We found that 19 genes showed up-regulated expression under blue light, including 4 MAPK and 15 MAPKKK genes, while 26 genes showed up-regulated expression under red light, including 6 MAPK, 1 MAPKK, and 19 MAPKKK genes. Two genes (*FtMAPKKK29* and *FtMAPKK38*) were not expressed in seedlings grown under the light.

Further analysis demonstrated that, in blue light, one Tartary buckwheat MAPK cascade gene was specifically up-regulated and eight genes were specifically down-regulated. Under red light, eight and one genes were specifically up-regulated and down-regulated, respectively. Influenced by combined blue light and red light, 18 and 36 Tartary buckwheat MAPK cascade genes were up- and down-regulated, respectively, indicating that, overall, the light had a negative regulatory effect on MAPK cascade gene expression in Tartary buckwheat.

From the perspective of gene expression changes, under blue light, *FtMAPKKK53* was most strongly up-regulated (5.30-fold), while under red light, the changes in MAPK cascade gene expression were not as marked as those observed under blue light, with *FtMAPK1* the most strongly up-regulated by only 2.25-fold.

**FIGURE 8:**
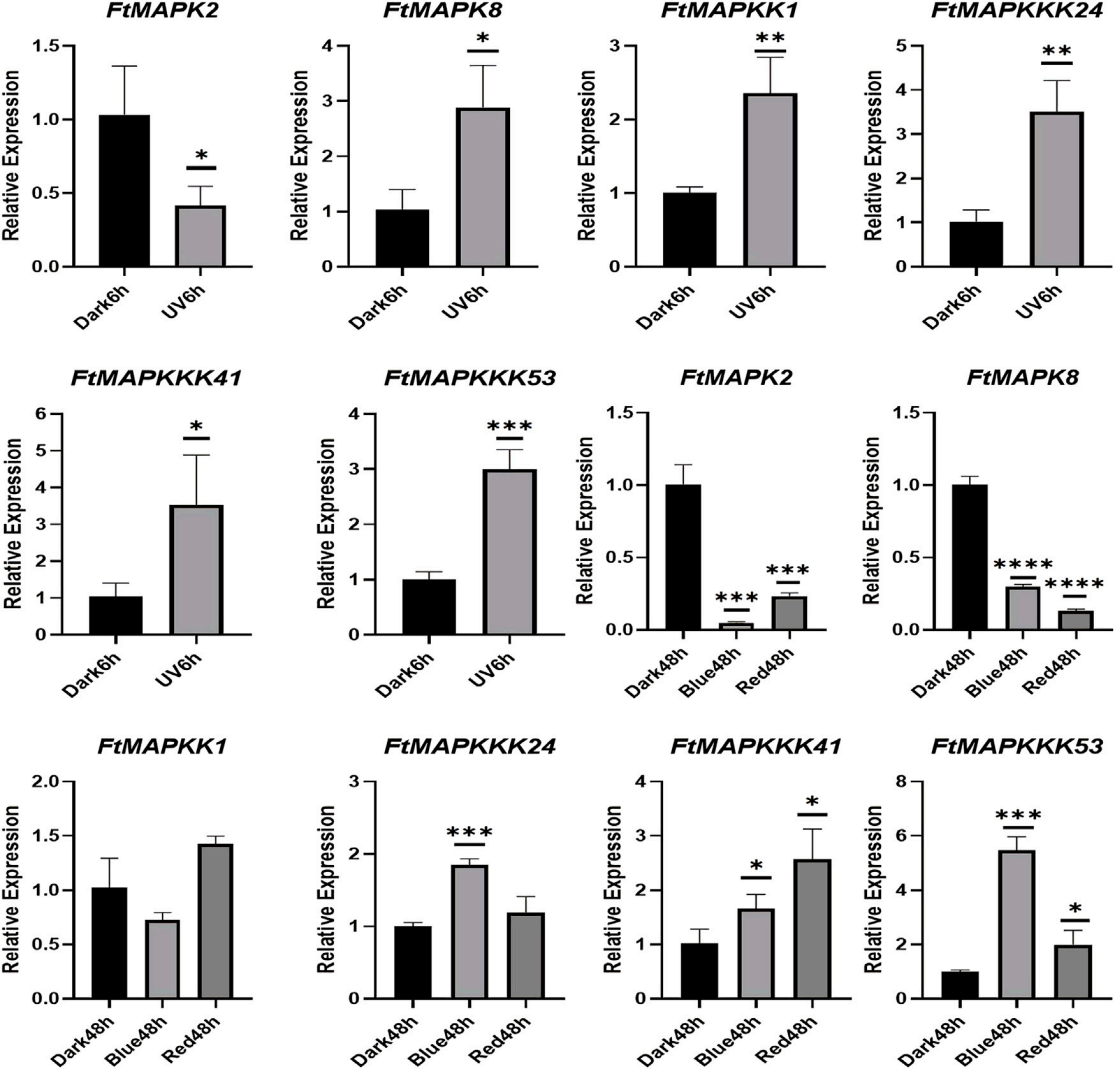
qRT-PCR analysis of 6 FtMAPK cascade genes in Tartary buckwheat seedlings. Error bars denote the standard deviation of three replicates (*p < 0.05, **p < 0.01, ***p < 0.001, ****p < 0.0001).

To confirm the reliability of the RNA-Seq data, we randomly selected 6 FtMAPK cascade genes and evaluated their expression patterns in Tartary buckwheat seedlings by qRT-PCR. Furthermore, Tartary buckwheat seedling experimental groups treated in 6-h darkness and 6-h UV-B irradiation were evaluated (Figure 8). The results demonstrated that the expression trends of the six genes under dark conditions relative to seedlings grown under different light conditions were consistent with those of heat-map analysis, indicating that the RNA-seq data were reliable. Under UV-B irradiation, *FtMAPK2* expression decreased, while levels of the other five genes increased. The results presented above were used for further analyses.

### Pattern of FtMAPK Cascade Gene Expression Under Abiotic Stress

**FIGURE 9:**
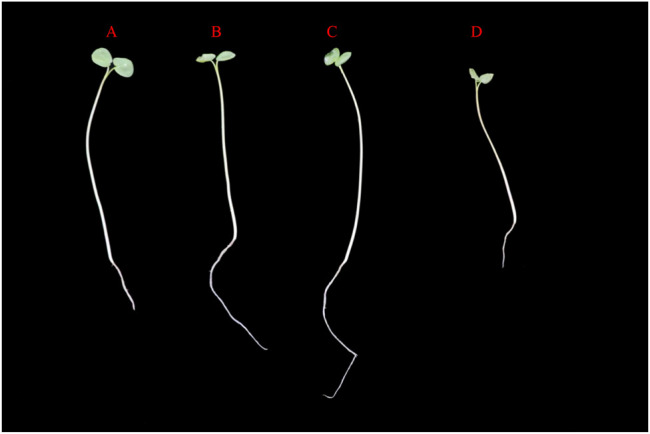
Growth status of Tartary buckwheat seedlings under abiotic stress. **(A)**: Tartary buckwheat seedlings grow under normal condition. **(B)**: Tartary buckwheat seedlings were treated with 100 mM for 24 h. **(C)**: Tartary buckwheat seedlings were treated with 20%PEG6000 for 24 h. **(C)**: Tartary buckwheat seedlings were treated at 40°C for 24 h.

MAPK cascade genes have been reported to help resist abiotic stress in numerous plants (Zhang et al., 2012). To investigate the expression pattern of MAPK cascade genes in Tartary buckwheat in response to abiotic stress, 4-day Tartary buckwheat seedlings were treated with high temperature (40 °C), drought (20% PEG6000), or salt (100 mM NaCl) stress for 24 h. In comparison with Tartary buckwheat seedlings grown under normal environmental conditions (CK), seedlings grown under drought and salt treatment had longer roots (Figure 9), with the roots of seedlings grown under drought treatment growing vigorously and longer, while the leaves were more smaller and the stems thinner. After high-temperature stress, the overall growth of seedlings deteriorated.

**FIGURE 10:**
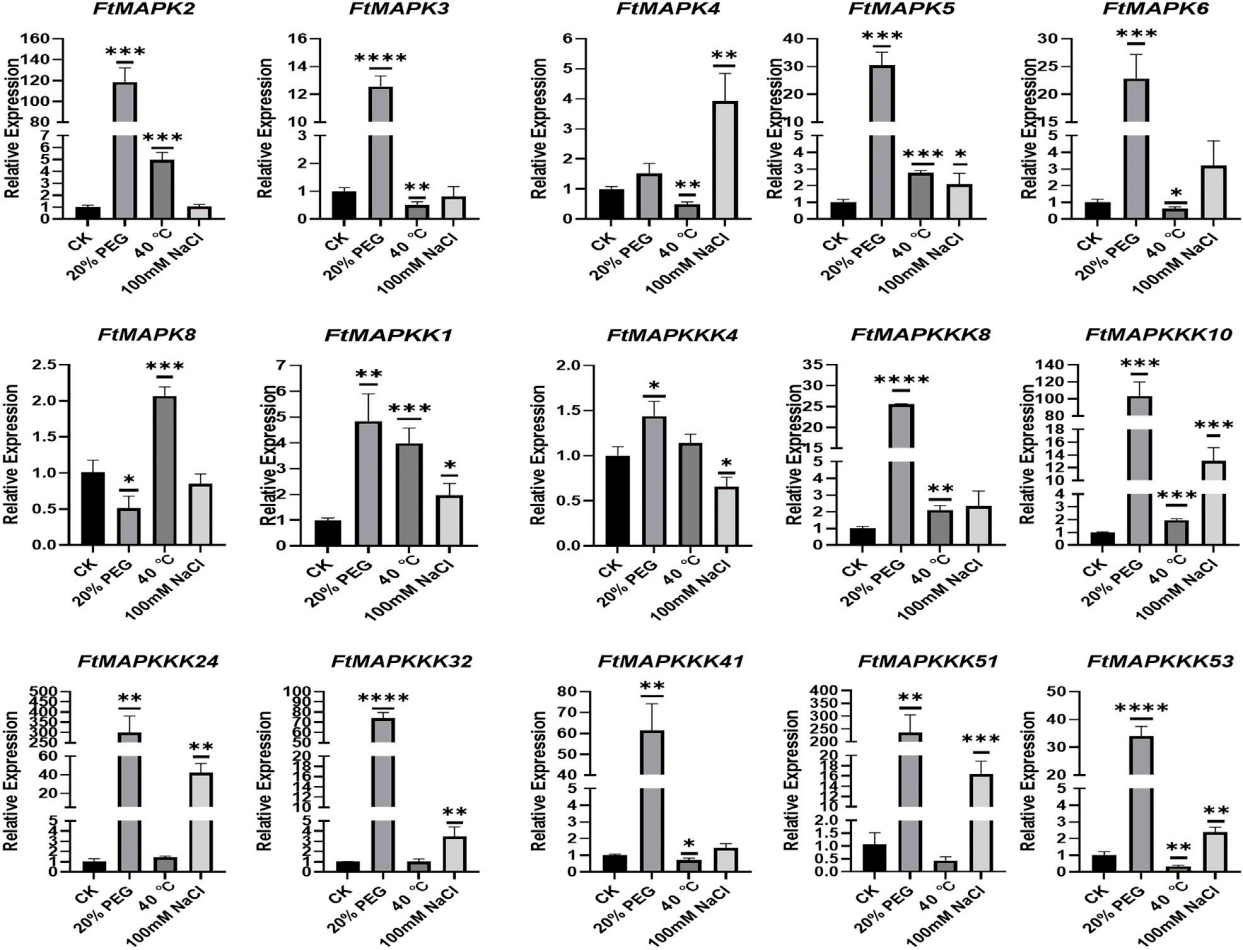
The expression profiles of FtMAPK genes under different abiotic stresses were performed three times per experiment. Error bars denote the standard deviation of three repeats (*p < 0.05, **p < 0.01, ***p < 0.001, ****p < 0.0001).

When plants encounter abiotic stress, their usual response is to increase the expression of stress-resistance genes (Mishra et al., 2020). To study the expression patterns of FtMAPK cascade genes under abiotic stress, a total of 15 genes were randomly selected from each subfamily for qRT-PCR analysis. The results showed that the expression of 14 genes, including all MAPKKK genes analyzed, responded strongly to drought stress, with their levels increasing significantly (Figure 10). Further, the expression levels of 11 genes increased under salt stress and levels of 11 genes were increased under high-temperature stress. Interestingly, the expression levels of *FtMAPK8* decreased under both drought and salt treatment, while they increased slightly under high-temperature stress. These results indicate that FtMAPK cascade genes may have dominant roles in an important function related to Tartary buckwheat responses to abiotic stress.

### Protein–Protein Interaction Network and GO Annotation Analysis of FtMAPK Cascade Molecules

**FIGURE 11:**
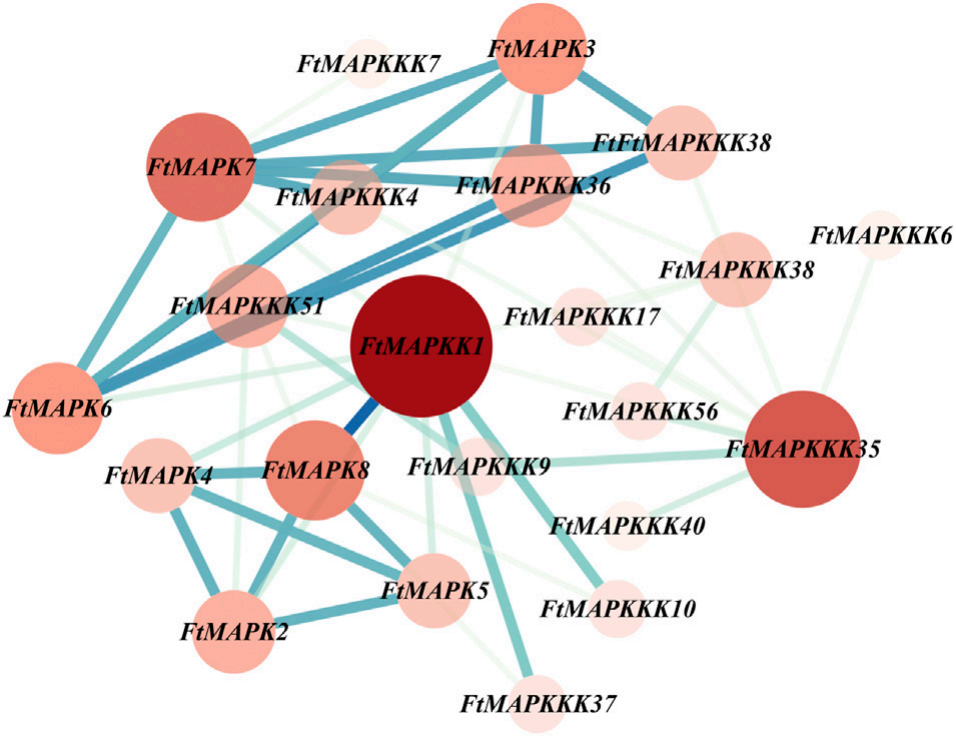
Tartary buckwheat MAPK cascade protein-protein interaction (PPI) network. The nodes represent proteins, the central node is represented by dark red, and the blue line represents the interaction between the nodes.It indicates the darker the color, the more important the protein is in the interaction network. The more nodes a node is connected to, the greater its degree value.

We employed the STRING database to predict the protein interaction characteristics of Tartary buckwheat MAPK cascade proteins; the results are presented in Figure 11, where nodes represent corresponding gene names and the degree value is represented by the size of the node and the depth of the color (Supplementary Table 6).

The whole PPI network consisted of 22 nodes and 48 edges, of which 9 proteins had degree values higher than the average. According to degree value, the top 5 proteins were: FtMAPK1, FtMAPKKK35, FtMAPK7, FtMAPK8, and FtMAPK3, and the proteins that ranked 6–9 were: FtMAPK6, FtMAPKKK36, FtMAPKKK51, and FtMAPK2. As shown in Figure 11, the FtMAPKK1, FtMAPKKK35, and FtMAPK7 proteins were located in the center of the PPI network, interacting with 12, 9, and 8 family member proteins, respectively. Therefore, we speculate that these three proteins have stronger interactions with other proteins and may have relatively important roles in plant growth and development.

### Identification and Analysis of Co-expression Transcription Factors With *MAPK* Cascade Genes

**FIGURE 12:**
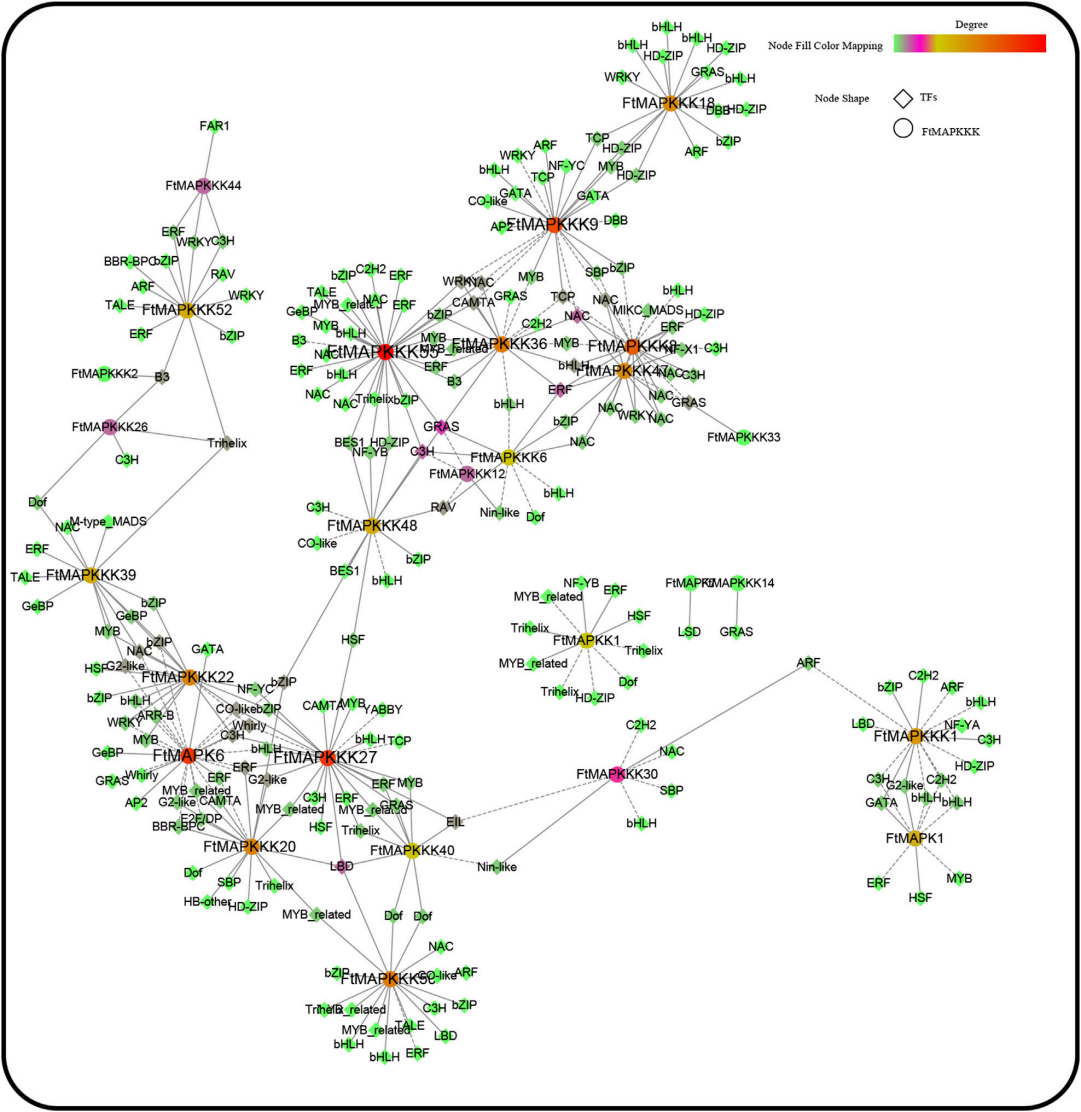
FtMAPK cascade co-expression network was constructed based on gene expression levels of Tartary buckwheat seedlings under different light conditions.

The online database, PlantTFDB (http://planttfdb.gao-lab.org/), was used to identify Tartary buckwheat transcription factors. A total of 1766 transcription factors were identified, among which the top 5 largest transcription factor families were: MYB (n = 175), bHLH (n = 164), ERF (n = 116), bZIP (n = 99), and NAC (n = 88) (Supplementary Table 7). Using python scripts, we conducted a co-expression analysis of Tartary buckwheat seeding gene expression under different light treatments, and transcription factors co-expressed with MAPK cascade genes (r > 0.9) (Tianyuan et al., 2017) were extracted and the results mapped (Figure 12). A total of 27 MAPK cascade genes were co-expressed with 40 transcription factors, of which the top 5 (largest numbers) transcription factors were NAC (n = 30), bHLH (n = 28), bZIP (n = 26), ERF (n = 26), and C3H (n = 19). Many studies have reported that these five types of transcription factors can help plants resist abiotic stress. Hence, when Tartary buckwheat encounters abiotic stress, FtMAPK cascade genes may work together with the identified transcription factors to resist adversity.

**FIGURE 13:**
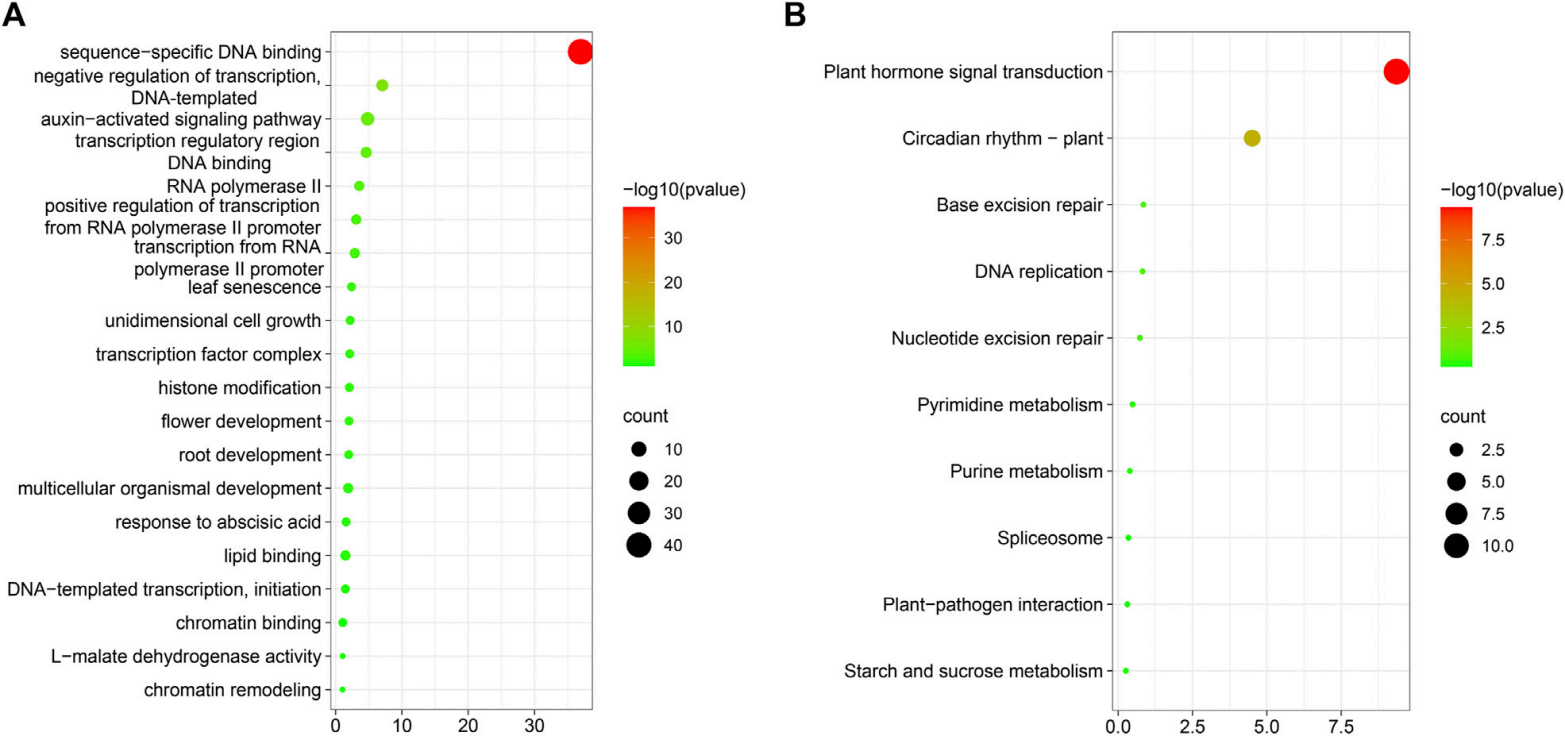
GO and KEGG enrichment analysis. **(A)**: Co-expression transcription factor GO enrichment analysis; **(B)**: Co-expression transcription factor KEGG enrichment analysis.

Next, we conducted GO and KEGG enrichment analyses of transcription factors identified as co-expressed with the 65 MAPK cascade genes (Supplementary Table 8). The main functions that emerged were sequence-specific DNA binding (Figure 13A), negative regulation of transcription, DNA-template, auxin-activated signaling pathway, transcription regulatory region DNA binding, RNA polymerase II transcription regulatory region sequence-specific DNA-binding transcription factor activity involved in positive regulation of transcription, positive regulation of transcription from RNA polymerase II promoter, and numerous other processes. Further, the proteins were involved in the promotion of seed germination, root development, leaf development, and flower development, during plant growth and development. Moreover, in the context of biotic and abiotic stress responses, they participate in many processes, such as salt stress, bacterial defense response, and auxin response.

Transcription factors respond through signal transduction into cells by regulating their target genes, thereby influencing cell biological processes. Plants are prompted to adapt to their environmental conditions through physiological mechanisms, such as DNA replication. The results of KEGG enrichment analysis (Figure 13B) showed that transcription factors co-expressed with MAPK cascade genes are mainly enriched in pathways involved in plant hormone signal transduction, gene replication, plant–pathogen interaction, and starch and sucrose metabolism, among others. In addition, plants are exposed to biotic and abiotic stresses.

**FIGURE 14:**
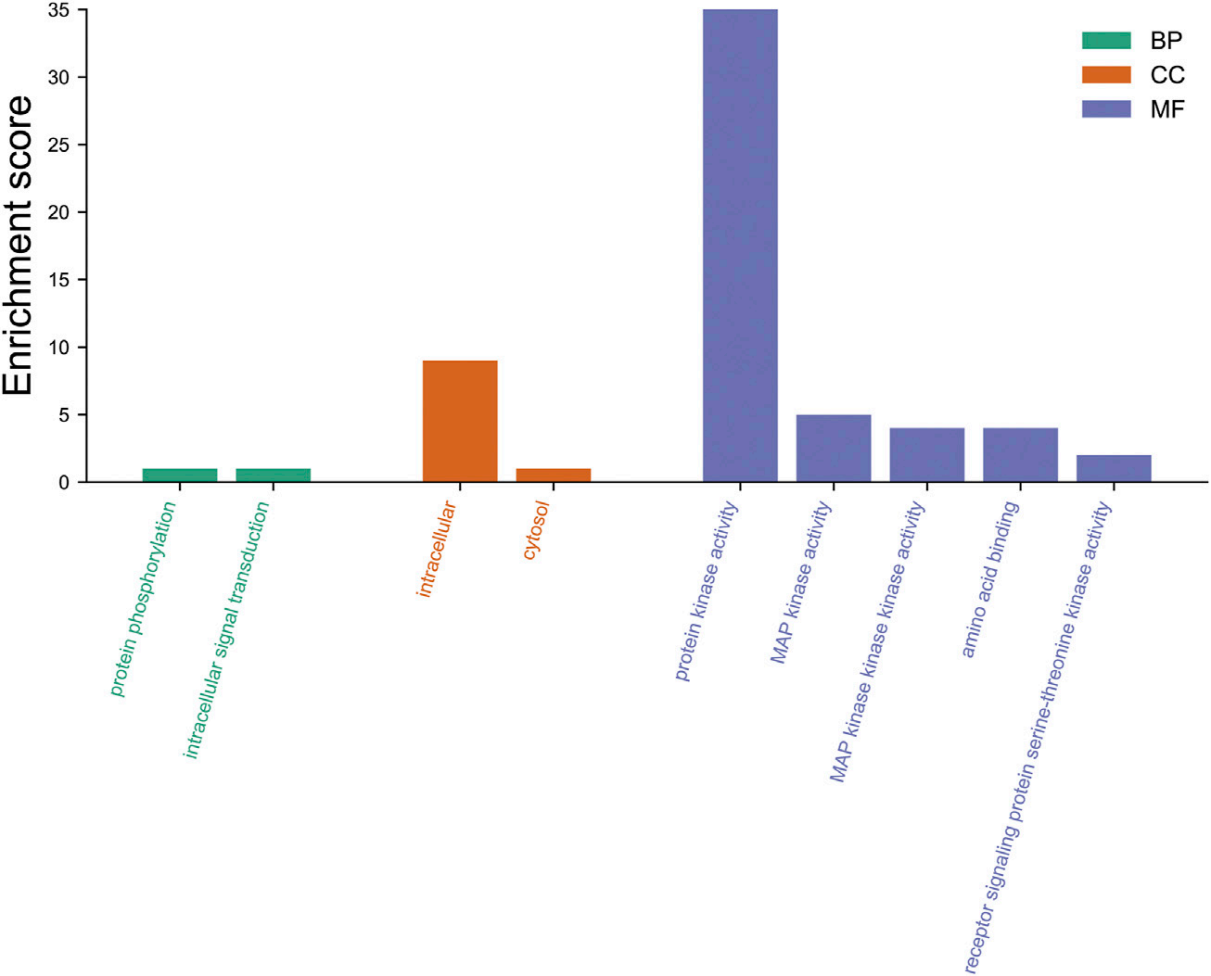
GO annotation of 65 FtMAPK cascade proteins. BP, biological processes; CC, cellular components; MF, molecular function.

We also annotated the biological function of FtMAPK cascade genes in Tartary buckwheat by using the eggNOG database (http://eggnog-mapper.embl.de/) to perform GO annotation of the 65 FtMAPK cascade genes (Figure 14). Our data demonstrated that the main biological processes enriched for these genes included protein phosphorylation and intracellular signal transduction. Annotation analysis of cellular components revealed that these proteins are mainly located intracellularly and in the cytosol, while their molecular function was primarily protein kinase activity. These results are consistent with the reported functions of MAPK cascade genes (Zhang et al., 2012).

## Discussion

MAPK cascade genes encode proteins involved in highly conserved signal transduction pathways that are ubiquitous in eukaryotes, including animals and plants. Further, MAPK cascade genes have important roles in regulating plant environmental adaptations, such as stress responses, osmotic regulation, and pathogenicity (Shi et al., 2009). A total of 65 MAPK cascade genes were identified in Tartary buckwheat and classified into three families: MAPK, MAPKK, and MAPKKK, with 8 in the MAPK family, the fewest MAPKK members (n = 1), and 56 MAPKKK genes. This distribution is consistent with that found in other plants; for example, there are 20, 10, and 80 MAPK, MAPKK, and MAPKKK genes, respectively, in *A. thaliana* (Colcombet and Hirt, 2008) (Alvarez-Flórez et al., 2013), and 12, 7, and 73 in *F. vesca* (Alvarez-Flórez et al., 2013) (Heying et al., 2017).

Gene expression responses to environmental changes may be slowed by introns, which influence the time from transcription to translation (Jeffares et al., 2008). In *Brachypodium distachyon*, MAPK genes have 3 to 11 introns, while 58.3% of MAPKK genes do not have introns (Chen et al., 2012). In *M. domestica*, the number of introns in MAPK family members ranges from 2 to 15, and there are no introns in 55.6% of MAPKK family members (Zhang et al., 2013). We discovered that the number of introns in Tartary buckwheat MAPK cascade genes was similar to that previously reported in other species, with MAPK family members containing 2–10 introns, no introns in the MAPKK family member, and a maximum of 16 introns in MAPKKK family members. Further, seven MAPK cascade genes did not contain introns. Simultaneously, we found both tandem and segmental duplication events involving FtMAPK cascade genes, with segmental duplication events (12 pairs) much more frequent than tandem duplication events (1 pair). Replication events contribute to gene function diversity, thereby improving the adaptability of plants to different environments (Kong et al., 2007); hence, their results indicate an essential role for MAPK cascade genes in plant evolution.

By constructing a phylogenetic tree using the model plant, *A. thaliana*, and mining conserved gene motifs, we evaluated the evolutionary relationships among Tartary buckwheat MAPK cascade genes and their degree of conservation during evolution. The Tartary buckwheat MAPK family can be divided into four subgroups: A, B, C, and D, consistent with their classification in *A. thaliana*, *S. lycopersicum*, and *M. domestica* (Yan et al., 2016). Additionally, there are special circumstances regarding the classification of the MAPK family genes in some plants; for example, those in grapes are divided into five subgroups (Birsen and Ozan, 2015), among which, group D has the largest number of genes. Arabidopsis MAPKK family genes are divided into four subgroups: A, B, C, and D, while only one MAPKK gene was identified in Tartary buckwheat, which was assigned to group C. This could be due to the combination or deletion of gene functions during evolution, leading to a decrease in the number of genes. Tartary buckwheat MAPKKK family genes are divided into three subgroups: RAF, MEKK, and ZIK, as observed in A. thaliana. Among MAPKKK family genes, the RAF subgroup contains the most genes, consistent with the results of the classification of MAPKKK family genes from other higher plants (Sinha et al., 2011). In addition, MAPK cascade gene–conserved motifs reported in numerous plants were also observed in FtMAPK. According to collinearity analysis, there were more collinear gene pairs in MAPK cascade genes in dicotyledons than in monocotyledons, consistent with the evolutionary relationship between dicotyledons and monocotyledons (Bin et al., 2021). The results presented above demonstrate that FtMAPK cascade genes have been conserved during evolution.

The production of secondary metabolites by plants is the result of their adaptation to an ecological environment during long-term evolution and is important for plant survival within ecosystems (Amec et al., 2021). Related reports have shown that MAPK cascade genes function in the regulation of plant flavonoid secondary metabolites; for example, Arabidopsis YODA (YDA), a member of the MAPKKK family, forms a complex by binding ETHYLENE-INSENSITIVE3 (EIN3), targets the Transparent Testa 8 (TT8) promoter, and forms a YDA-EIN3-TT8 cascade module to regulate anthocyanin biosynthesis (Meng et al., 2018). The R2R3 MYB transcription factor, AtMYB75, interacts with AtMAPK4, and AtMYB75 is phosphorylated by AtMAPK4 to achieve its full function, which involves participating in light-induced anthocyanin accumulation in Arabidopsis (Li et al., 2016). In a previous research study, our team found that rutin, astragalin, quercetin, isorhamnetin-3-O-glucoside, isorhamnetin, and trifolin increase in Tartary buckwheat seedlings under the influence of blue and red light, based on transcriptome and qRT-PCR data analyses (Zhang et al., 2018). Here, we found that 18 *FtMAPK* cascade genes were up-regulated in response to red and blue light, and we predict that they may be involved in regulating flavonoid secondary metabolite synthesis in Tartary buckwheat seedlings.

We subjected Tartary buckwheat to three types of abiotic stress and performed qRT-PCR experiments on 15 selected *FtMAPK* cascade genes to identify functional molecules involved in abiotic stress resistance. It is established that MAPK cascade genes regulate plant physiological responses to abiotic stress. In this analysis, we found that MAPK cascade genes all contained *cis-*elements involved in defense and stress responses. MAPK cascade genes play important roles in the responses of higher plants to drought stress. For example, overexpression of the *M. domestica* RAF MAPKKK family subgroup member, *MdRaf5*, in A. thaliana strengthens the drought resistance of plants by reducing transpiration rate and stomatal pore size (Sun et al., 2017). In *F. vesca*, the B subgroup MAPK family genes, *FvMAPK5* and *FvMAPK8*, are transcriptionally activated by drought (Zhou et al., 2017). In Tartary buckwheat, expression levels of the RAF subgroup *FtMAPKKK4*, *FtMAPKKK24*, *FtMAPKKK32*, and *FtMAPKKK53* genes, and *FtMAPK4* and *FtMAPK5* belonging to the B subgroup of the MAPK family, were also significantly increased under drought stress, indicating that they have similar functions in resisting drought stress.

Land salinization is a major environmental factor that influences plant growth (Cui et al., 2013), by causing yellowing, wilting, and severe yield reduction (Zhang et al., 2020). There is increasing evidence that MAPK cascade genes are key regulators of responses to salt stress in higher plants. For example, overexpression of *Chenopodium album CaMKK1* in tobacco can effectively eliminate reactive oxygen species and enhance plant tolerance to salt stress (Wang et al., 2017). After treatment of four-week-old *Actinidia chinensis* seedlings with high salt, expression levels of *AcMAPK4*, *AcMAPK5*, *AcMAPK9*, and *AcMAPK12* were significantly up-regulated at all treatment time points (Gang et al., 2018). In 14-day Chrysanthemum morifolium seedings, salt stress induced specific high expression of *CmMPK13* and *CmMKK4* in roots (Aiping et al., 2018); similarly, the expression levels of *FtMAPK4*, *FtMAPK6*, *FtMAPK5*, and *FtMAPKK1*, encoding MAPK and MAPKK family proteins, were increased under salt stress.

With changes in the Earth’s climate, high temperature has become an important factor affecting the growth and development of major food crops. Current research results show that MAPK cascade genes function in regulating the tolerance of higher plants to heat stress. For example, when Arabidopsis is under heat stress, HSP90 (heat-shock protein 90) interacts with YODA to activate the downstream AtMKK4/5–MPK3/6 cascade pathway and adapts to the thermal environment by controlling the development of stomata (Samakovli et al., 2020). In *F. vesca*, transcription levels of *FvMAPK3*, *FvMPKK1*, *FvMPKK3*, *FvMPKK6*, and *FvMPKK7* are significantly up-regulated under high-temperature treatment. Additionally, the MAPK genes in mulberry are also involved in responses to extreme temperatures. After high-temperature treatment (40 °C), the expression levels of eight *MnMAPK* genes were significantly up-regulated (Wei et al., 2014). In this study, we found that *FtMAPK5*, *FtMAPK8*, and *FtMAPKK1* genes in the MAPK and MAPKK families were up-regulated after treatment at 40 °C, with FtMAPK8 specifically responding to high-temperature stress. These results demonstrate that *FtMAPK* cascade genes actively participate in Tartary buckwheat responses to abiotic stress.

MAPK cascade pathways are activated by sequence-specific phosphorylation. MAPKKK genes encode the most upstream kinases in MAPK cascades and are activated by phosphorylation in response to sensing external stimuli. Then, MAPKKK proteins phosphorylate MAPKKs to complete MAPKK activation. As the most downstream molecules of the cascade, MAPK proteins enter the nucleus after they are phosphorylated and activated by MAPKK, and induce functional gene expression by activating specific transcription factors (Liu et al., 2015). Combined analysis of the qRT-PCR data generated under different stress conditions and MAPK protein–protein interaction networks indicated that FtMAPKKK4, FtMAPKK1, and FtMAPK6 undergo protein–protein interactions. Under abiotic stress, the expression of these genes increases, and they have upstream *cis-*elements involved in defense and stress responses. The *MAPKK1* gene also contains an element that is bound by MYB and participates in regulating flavonoid synthesis. Therefore, we predict that, when Tartary buckwheat is subjected to abiotic stress, it activates the FtMAPKKK4–FtMAPKK1–FtMAPK6 pathway, and combines with MYB transcription factors to increase flavonoid synthesis, thereby reducing the damage to plants caused by abiotic stress.

In the present study, a total of 27 MAPK cascade genes were found to have co-expression relationships with 40 transcription factors, with the top 5 types of transcription factors including those from the NAC (Shipeng et al., 2021), bHLH (Mao et al., 2017), bZIP (Nijhawan et al., 2008), ERF (Han et al., 2021), and C3H (Xinran Cheng, 2020) families. These gene families are involved in plant stress responses. When Tartary buckwheat is subjected to abiotic or biological stress, it can activate MAPK cascade pathways by sensing external stress signals, and subsequently regulate transcription factors with anti-stress functions. Thus, Tartary buckwheat can adapt to growth in different environments, and research to understand MAPK cascade pathways is important and valuable for understanding the growth and development of Tartary buckwheat.

## Conclusion

To conclude, here we conducted a systematic study of the Tartary buckwheat FtMAPK cascade gene family. We identified 65 FtMAPK cascade genes and classified them into three families: MAPK, MAPKK, and MAPKKK by constructing a phylogenetic tree with the model plant, *A. thaliana*. These genes are distributed on five chromosomes. Analysis of gene expression in Tartary buckwheat seedlings under three abiotic stress conditions demonstrated that levels of FtMAPK5, FtMAPKK1, FtMAPKKK8, FtMAPKKK10, and FtMAPKKK24 were increased. Combined with protein–protein interaction network analysis, allowed the prediction of Tartary buckwheat responses to abiotic stress through MAPK cascade pathways. We also constructed a co-expression network of FtMAPK cascade genes and annotated the functions of related transcription factors. Together, these data indicate that FtMAPK cascade genes have important roles in the growth and development of Tartary buckwheat and in responses of this plant to abiotic stress.

## Data Availability

All data generated or analyzed during this study are included in this published article and its supplementary information files. The raw sequencing data used during the study have been deposited in NCBI SRA with the accession number SRP157461.

## Supplementary Material

Supplementary Table 1 The list of 65 *FtMAPK* cascade genes identified in this article.

Supplementary Table 2 Phylogenetic analysis of Tartary buckwheat MAPK cascade protein.

Supplementary Table 3 Chromosomal locations and distribution directions of *FtMAPK* cascade genes.

Supplementary Table 4 The list of *cis*-elements of *FtMAPK* cascade genes promoter.

Supplementary Table 5 Expression data of *FtMAPK* genes in Tartary buckwheat seedlings under different lighting treatments.

Supplementary Table 6 The list of FtMAPK cascade protein and protein interaction.

Supplementary Table 7 List of transcription factors co-expressed with FtMAPK cascade genes.

Supplementary Table 8 GO and KEGG enriched annotation information.

Supplementary Table 9 The primers sequences used in this study.

Supplementary Table 10 The qRT-PCR’s raw data.

## Abbreviations

MAPK, mitogen-activated protein kinase; MCScanX, Multiple Collinearity Scan toolkit; PPI, Protein–protein interaction; KEGG, Kyoto Encyclopedia of Genes and Genomes databases.
